# A Meta-Analysis of Vascular Endothelial Growth Factor for Nasopharyngeal Cancer Prognosis

**DOI:** 10.3389/fonc.2018.00486

**Published:** 2018-10-31

**Authors:** Feng Wang, Lisha Peng, Yong Wang, Xiaodong Liu

**Affiliations:** ^1^Key Laboratory of Radiobiology, Ministry of Health, School of Public Health, Jilin University, Changchun, China; ^2^Department of Radiotherapy, The First Affiliated Hospital of Kunming Medical University, Kunming, China

**Keywords:** vascular endothelial growth factor (VEGF), nasopharyngeal cancer (NPC), survival, prognosis, meta-analysis

## Abstract

**Background:** Vascular endothelial growth factor (VEGF) has been reported to serve as a promising prognostic marker in several cancers. This meta-analysis aims to assess the prognostic significance of VEGF in nasopharyngeal cancer (NPC).

**Methods:** We conducted a systematic literature search of PubMed, Embase, and the Cochrane Library for observational studies published until June, 2018 to identify observational studies on the prognostic effect of tissue VEGF expression or serum VEGF level on the survival of NPC. The primary outcome measure assessed was overall survival (OS). The secondary outcomes included disease-free survival (DFS) or progression-free survival (PFS). Summary hazard ratio (HR) and its 95% confidence interval (95% CI) were derived using a random-effects model.

**Results:** Out of 840 retrieved citations, 16 studies inclusive of 1,345 patients were included in the analysis of tissue VEGF expression and cancer survival. The pooled HRs for OS and DFS in patients with high VEGF expression were 2.07 (95% CI: 1.32–3.25) and 5.99 (95% CI: 2.66–13.48), respectively, with significant heterogeneity between studies (I^2^ = 79.1% for OS and 50.2% for DFS). Tissue high VEGF expression was not significantly associated with short RFS, PFS, or MFS. Five studies also investigated the prognostic effect between serum VEGF level and patient survival and found that high serum VEGF level was significantly associated with short OS for patients with NPC (HR 2.47, 95% CI 1.16–5.28), but not with short PFS (HR 1.47, 95% CI 0.92–2.35).

**Conclusions:** Determination of tissue VEGF expression and serum VEGF level have the potential to serve as biomarkers and add prognostic information in NPC. Prospective analyses of associated data on VEGF expression and serum VEGF level in large NPC cohorts could be further conducted to advance our understanding of the relationship between VEGF and NPC outcomes.

## Introduction

Nasopharyngeal cancer (NPC), a rare cancer with unbalanced distribution, has a high prevalence rate in southern China, with 85,000 estimated new cases and 50,000 estimated deaths worldwide in 2012 (1). Due to deep location in the nose and to its non-specific initial clinical manifestation, NPC is often diagnosed at an advanced stage with a 5-year overall survival (OS) rate below 40% (2). As a distinct entity among head and neck cancers, NPC has been largely demonstrated that several clinico-pathological factors affect patients' survival, including patient age, gender, pretreatment serum EBV DNA level, tumor stage, primary cancer volume, sensitivity to radiotherapy, and chemotherapy (3–5). It may also be worth mentioning that the biology of this tumor appears to vary widely between endemic (predominantly Asian) and non-endemic areas.

Nevertheless, recent advancement achieved in the field of NPC is that it involves several associated signaling pathways contributing to the biological and clinical behavior of NPC, one of which was JAK2/STAT3 signaling pathway (6). This pathway has been reported to be involved in multiple cellular functions such as differentiation, survival, proliferation, and apoptosis (7, 8). STAT3 possesses regulatory abilities in angiogenesis through the transcription of vascular endothelial growth factor (VEGF) (9), a potent angiogenic factor which plays a crucial role in a several pathological processes including microvascular permeability, tumor cell penetration (6), acting as an important mediator of angiogenesis, representing a potential target for anticancer therapy (10).

Recently published studies and meta-analyses indicated that VEGF is a promising prognostic biomarker for papillary thyroid cancer (11), oral tongue squamous cell carcinoma (12), cervical cancer (13, 14), colorectal cancer (15), and other cancers (16–27). Regardless of various issues existing in the published literature in terms of the PROGRESS series in the field of prognosis research and the reporting of prognostic studies of tumor markers (REMARK guidelines) (28, 29), the prognostic value for NPC setting is still in debate (12), thus leading to the need of clarifying the role of this molecular target in predicting NPC patients' survival outcome. Therefore, we aim to assess the value of VEGF as a prognostic factor in NPC.

## Materials and methods

### Search strategy

This meta-analysis was performed in accordance with the Meta analysis of Observational studies in Epidemiology (MOOSE) guidance (30). We conducted a systematic literature search of PubMed, Embase, and the Cochrane Library for observational studies published until June, 2018 to investigate the prognostic effect of tissue VEGF expression or serum VEGF level on the survival of NPC using the strategy shown in Supplementary Material. We read through all of the generated citations using the search strategy and selected the associated full text of selected publications to determine the final included studies satisfying the inclusion criteria. We also hand searched references from the included studies to identify additional studies which could be missed during literature search and selection. We did not apply language restrictions during the literature search. A Google Scholar search using similar keywords was also conducted for gray literature.

### Study selection and inclusion criteria

At least two investigators (FW and LP) read all titles or abstracts of identified citations through database search. The selected relevant articles were then cross checked by two independent investigators for possible inclusion and disagreements were resolved through discussion or by consensus with a senior investigator (XL).

Prospective or retrospective observational studies fulfilling the following inclusion criteria were included in further analysis: studies in humans with clinically or pathologically diagnosed NPC reporting the effect of tissue VEGF expression or serum VEGF level on patient survival were included; publications evaluating VEGF expression using immunohistochemistry (IHC) in human tissues from primary NPC or investigating plasma VEGF level with enzyme-linked immunosorbent assay (ELISA) before treatment; studies analyzing the association between VEGF overexpression with at least one of the following survival outcomes overall survival (OS), disease-free survival (DFS), recurrence-free survival (RFS), metastasis-free survival (MFS), or progression-free survival (PFS). Studies were excluded if only RNA data were analyzed, or having small sample size of <30 patients. Studies were also excluded if they used duplicated samples from the same study cohort.

### Data abstraction

Data were extracted by two investigators (FW and LP) into a predesigned spreadsheet and cross-checked by each other. The following summarized variables were collected from each study: first author, publication year, research country, the number of cases, cancer stage, follow-up period, sample origin, VEGF assay, cut-off level, statistical method, study quality score, and outcome investigated.

### Assessment of bias

Observational studies for cohort or case-control studies were assessed for bias using a 9-star Newcastle-Ottawa Scale (NOS) (31). A final NOS score was obtained in terms of selection of the involved population, comparability of study groups, and adequacy of outcome assessment. We defined that a score of 6 or below was considered high risk of bias (low study quality); and a score of 7 or above was considered low risk of bias (high study quality) (32, 33). Conflicts were resolved by joint discussion. We also applied reporting recommendations for tumor marker prognostic studies (REMARK) to evaluate study quality in cancer-related meta-analyses based on Supplementary Table 1 (34).

### Outcome measures

The primary outcome was OS, defining as the proportion of patients who did not die from any cause. The secondary outcomes included DFS/RFS/MFS/PFS, defined as the proportion of patients free from any disease/local recurrence/metastatic recurrence/disease progression.

### Statistical analysis

Meta-analysis was performed with Stata® version 12.0 (Stata Corp LP, College Station, Texas, USA). Because of the clinical heterogeneity inherent in our study, we applied random effects models for all meta-analyses (35). For adjusted or unadjusted HRs, we used the inverse variance method. Statistical heterogeneity was assessed with I^2^ statistic (36). To further explore heterogeneity, we performed *post-hoc* subgroup analyses for OS subset stratified by study characteristics such as study region, sample size, IHC cut-off level, statistical method, and the risk of bias. We assessed publication bias by visual inspection of funnel plot asymmetry, Begg's rank correlation test (37) and Egger's linear regression test (38), with a *P*-value < 0.1 indicating existence of publication bias. To further test the robustness of the main findings, we also performed sensitivity analyses by omitting one study at a time and recalculating the others.

## Results

### Literature search

**Figure 1:**
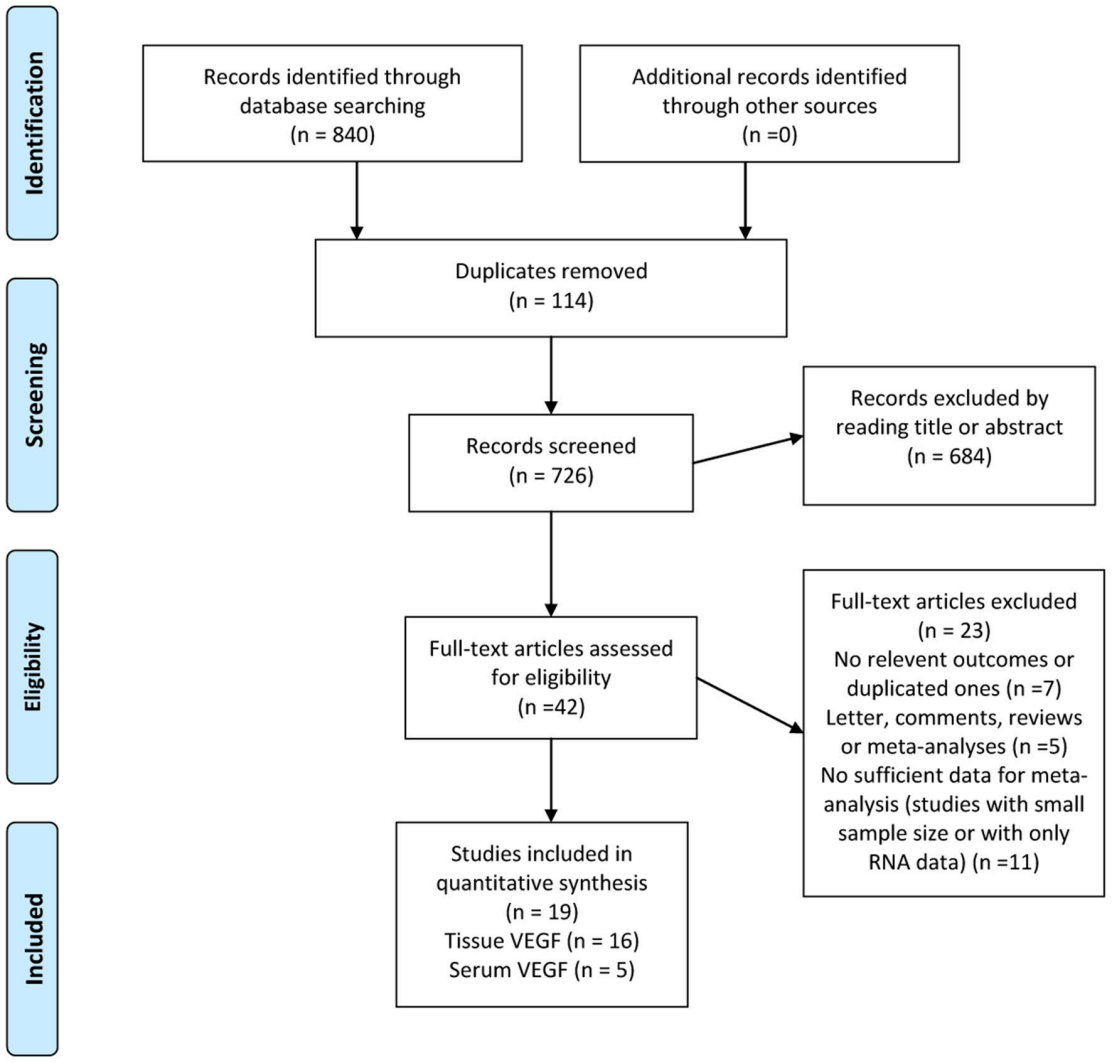
Selection process for studies to be included in the meta-analysis in compliance with MOOSE (Meta analysis of Observational studies in Epidemiology) standards.

The search strategy yielded a total of 840 studies, of which data from 19 studies were used, comprising 1,840 patients (Figure 1) (39–57). Of these, 16 studies had reported the prognostic value of tissue VEGF expression in NPC and 5 studies had evaluated serum VEGF level in NPC (53–57).

### Study demographics

The demographics of each included study are presented in detail in Table 1. In summary, all these studies were conducted in single clinical centers and were published between 2001 and 2018. Seventy-four percent of studies analyzed patients from China, 21% from other Asian countries, 5% from the USA, and 32% of studies analyzed data from more than 100 patients. The majority of the studies included patients with all stages of cancers, except one included early cancers and one advanced cancers. The mean/median follow-up period ranged from 16 to 116 months and 3 studies did not provide the mean/median follow-up period.

**Table 1:** Baseline characteristics of included studies.

**Author (year)**	**Country**	**Cases**	**Cancer stage**	**Follow-up (mean)**	**Sample**	**VEGF assay**	**Cut-off**	**Field**	**Statistical method**	**NOS/REMARK item score**	**Outcome investigated**
Cheng et al. (52)	China	50	I–IV	Mean 32.6 months	Tissue	IHC	Score	400×	M	8/14	OS
Zhang et al. (40)	China	96	I–IV	Mean 3 years	Tissue	IHC	>5%	NR	KM	7/13	OS
Liang et al. (54)	China	57	III–IV	Mean 5 years	Serum	ELISA	>455.61 ng/L	NR	Km	6/12	RFS
Pan et al. (47)	China	128	II–IV	Median 116 months	Tissue	IHC	>25%	NR	M	8/16	OS/DFS/ RFS/MFS
Kim et al. (50)	Korea	69	I–IV	Median 54 months	Tissue	IHC	Score	200×	M	8/15	OS
Lv et al. (53)	China	306	I–IV	>36 months	Serum	ELISA	>387.0 ng/L	NR	KM	6/12	OS/MFS
Chang et al. (57)	China	132	I–IV	NR	Serum	ELISA	>14.4 pg/mL	NR	KM	5/12	OS
Kurnianda et al. (55)	Indonesian	30	III–IV	Median 16 months	Tissue/ serum	IHC/ELISA	>25%/≥ 834 pg/ml	NR	KM	7/13	PFS
Segawa et al. (45)	Japan	76	I–IV	Median 28.9 months	Tissue	IHC	Score	NR	KM	7/12	OS
Li et al. (48)	China	188	I–IV	NR	Tissue	IHC	>10%	100×	M	8/14	OS
Xueguan et al. (42)	China	59	II–IV	Median 63 months	Tissue	IHC	>10%	200×	M	7/15	OS/PFS
Zhao et al. (39)	China	66	I–IV	Median 41 months	Tissue	IHC	>5%	400×	KM	5/14	OS
Parikh et al. (46)	USA	106	I–II	Median 9.9 years	Tissue	IHC	>25%	NR	M	7/15	OS/PFS
Sha and He (44)	China	127	I–IV	Median 67.5 months	Tissue	IHC	Score	200×	KM	4/13	OS
Krishna et al. (49)	India	64	I–IV	NR	Tissue	IHC	>25%	400×	KM	5/12	OS
Shi et al. (43)	China	62	I–IV	>3 years	Tissue	IHC	>10%	400×	KM	4/9	OS
Guo et al. (56)	China	59	I–IV	Median 36.3 months	Tissue/ serum	IHC/ ELISA	>25% /466.78 ng/L	400×	M	6/14	PFS
Hui et al. (51)	China	90	II–IV	Median 4.13 years	Tissue	IHC	>25%	400×	KM	6/14	OS/ PFS
Zhang et al. (41)	China	75	I–IV	>4 years	Tissue	IHC	>10%	400×	KM	5/13	OS

*ELISA, enzyme linked immunosorbent assay; IHC, immunohistochemistry; KM, Kaplan–Meier method; M, multivariate analysis; MFS, metastasis-free survival; NOS, Newcastle-Ottawa Scale; OS, overall survival; PFS, progression-free survival; REMARK, REporting recommendations for tumor MARKer prognostic studies; VEGF, vascular endothelial growth factor*.

### Survival analysis

**Figure 2:**
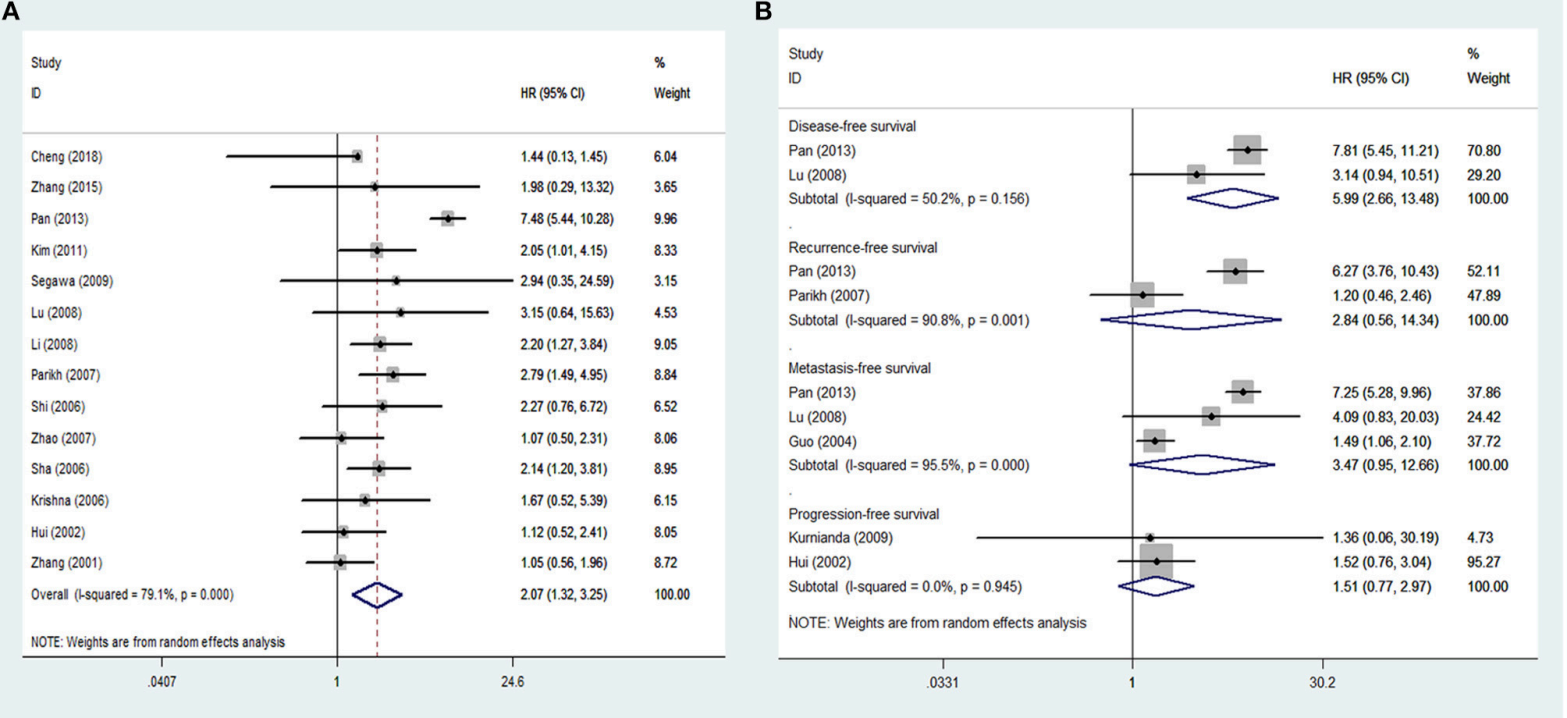
Meta-analysis of tissue VEGF expression for **(A)** overall survival; **(B)** disease-free survival, recurrence-free survival, metastasis-free survival and progression-free survival in nasopharyngeal cancer. Weights are from random effects analysis. CI, confidence interval; HR, hazard ratio; W (random), Weights (random effects model).

Sixteen studies inclusive of 1,345 patients were included in the analysis of tissue VEGF expression and cancer survival. The pooled HR for OS in patients with high VEGF expression compared with low expression was 2.07 (95% CI: 1.32–3.25), with a significant degree of heterogeneity (I^2^ = 79.1%) (Figure 2A), while the pooled HR for DFS was 5.99 (95% CI: 2.66–13.48), again with a high degree of inter-study heterogeneity (I^2^ = 50.2%) (Figure 2B). The pooled HRs for RFS and MFS, which were only analyzed in two and three studies, respectively, were 2.84 (95% CI: 0.56–14.34) and 3.47 (95% CI: 0.95–12.66), again both with high inter-study heterogeneity (I^2^ = 90.8% for RFS and 95.5% for MFS) (Figure 2B). The pooled HRs for PFS, which was analyzed in two studies, was 1.51 (95% CI: 0.77–2.97), with no inter-study heterogeneity (I^2^ = **0**).

### Subgroup analyses

We conducted several subgroup analyses based on study region, sample size, follow-up period, VEGF expression cut-off value, VEGF expression criteria (high vs. low and positive vs. negative) and NOS score. We noted that the results of subgroups remained similar to that of the main analysis. Furthermore, the I^2^ statistics significantly decreased in half of the subgroups, indicating part of the heterogeneity could result from these factors (Table 2).

**Table 2:** Subgroup analyses for associations between tissue VEGF expression and overall survival for patients with nasopharyngeal cancer.

**Sratified variables**	**HR**	**95% CI**	**Heterogeneity (I^2^ statistics; %)**	**No. of included studies**	***P* for interaction**
Total	2.07	1.32–3.25	79.1	14	NA
**STUDY REGION**					0.299
Endemic regions	1.99	1.09–3.62	85.0	10	
Non-endemic regions	2.350	1.55–3.57	0	4	
**SAMPLE SIZE**					< 0.001
≥100	3.23	1.58–6.60	87.9	4	
< 100	1.44	1.07–1.94	0	10	
**FOLLOW-UP PERIOD (MEAN/MEDIAN)**					< 0.001
< 5 years	1.52	1.05–2.19	0	7	
≥5 years	2.74	1.20–6.26	89.6	5	
**VEGF EXPRESSION CUT-OFF VALUE**					< 0.001
>5–10%	1.58	1.12–2.23	4.1	6	
>25%	2.65	1.02–6.91	89.1	4	
Score	2.04	1.35–3.07	0	4	
**VEGF EXPRESSION CRITERIA**					< 0.001
High vs. low	2.19	0.77–6.21	92.7	4	
Positive vs. negative	1.93	1.47–2.54	0	10	
**NOS SCORE**					< 0.001
< 7	1.43	1.05–1.95	0	6	
≥7	2.85	1.62–5.03	74.5	8	

*CI, confidence interval; HR, hazard ratio; NOS, Newcastle-Ottawa Scale*.

**Figure 3:**
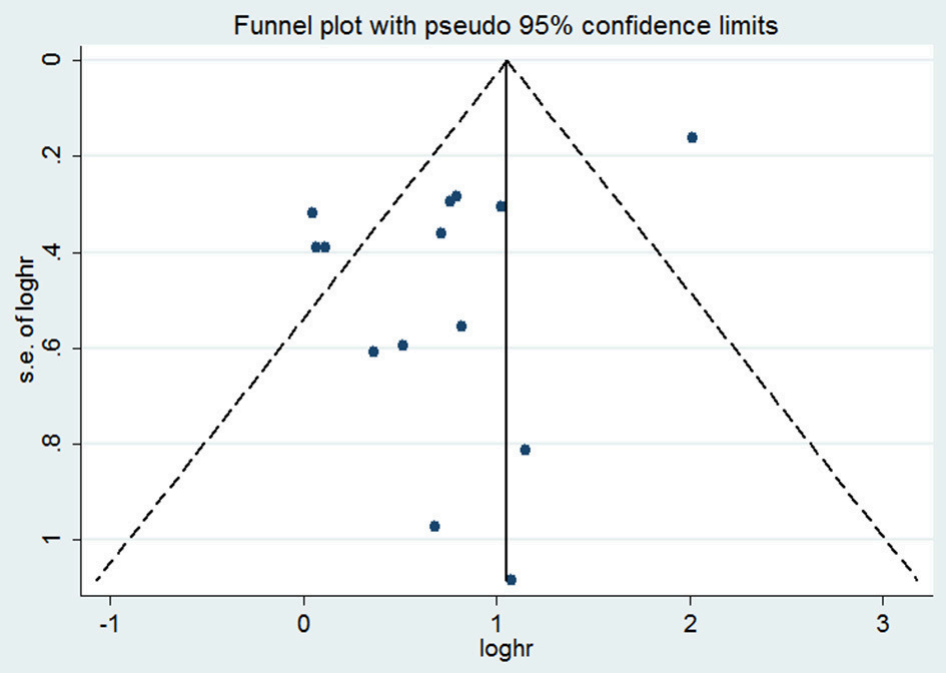
Funnel plot with tissue VEGF expression for overall survival. HR, hazard ratio; s.e., standard error.

Funnel plots for OS demonstrated certain evidence of publication bias by Egger's test (*P* = 0.031) (Figure 3). However, the results did not change after applying the trim-and-fill method, indicating the robustness of the summary result. We did not test publication bias for outcomes of DFS, RFS, MFS, or PFS due to limited number of studies. Sensitivity analyses by omitting one study at a time and recalculating the summary HRs for the remaining ones obtained similar results.

**Figure 4:**
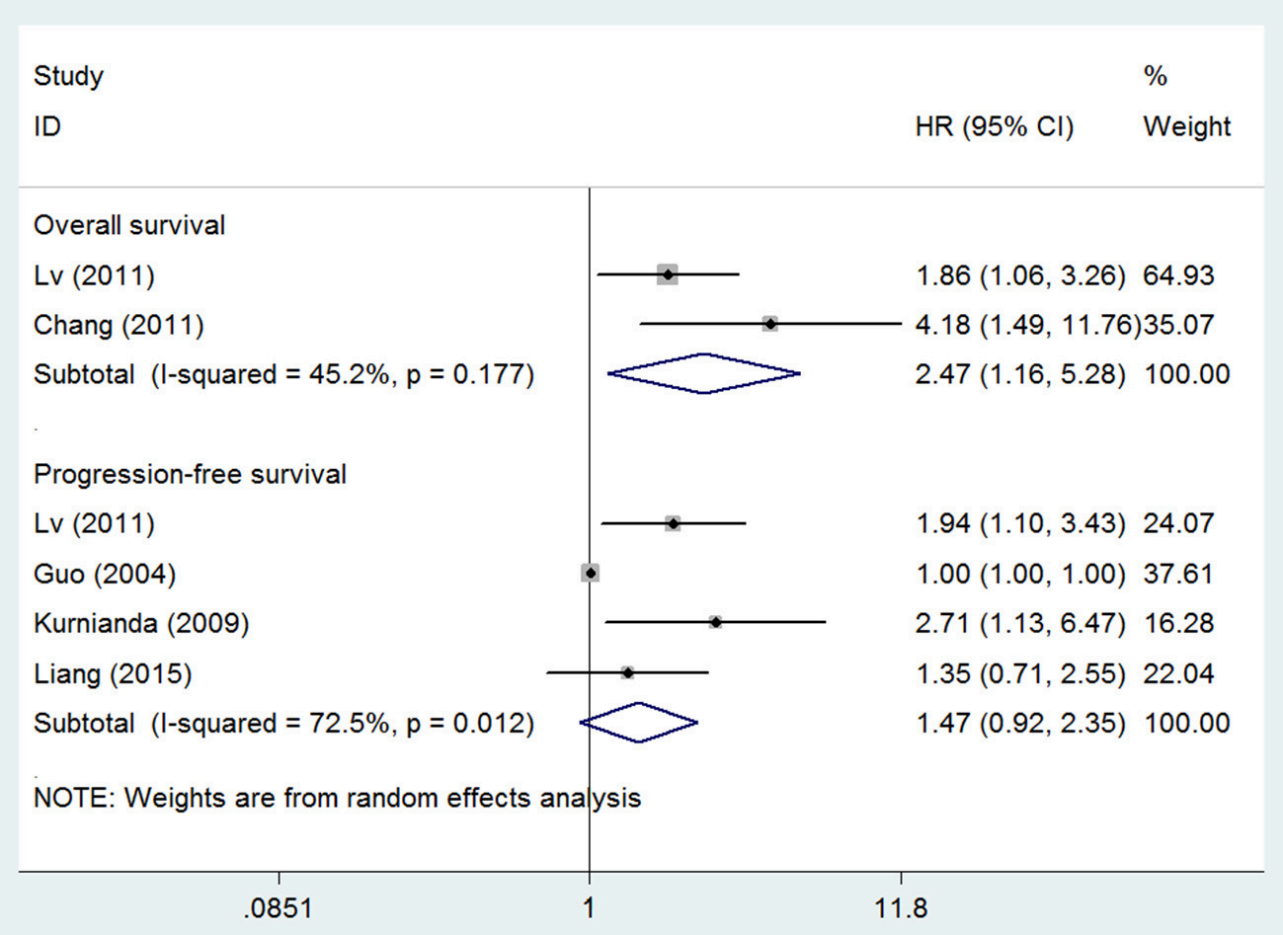
Meta-analysis of serum VEGF level for overall survival and progression-free survival in nasopharyngeal cancer. Weights are from random effects analysis. CI, confidence interval; HR, hazard ratio; W (random), Weights (random effects model).

Five studies also investigated the prognostic effect between serum VEGF level and patient survival and found that high serum VEGF level was significantly associated with short OS for patients with NPC (HR 2.47, 95% CI 1.16–5.28), but not with short PFS (HR 1.47, 95% CI 0.92–2.35) (Figure 4). We also did not test publication bias for these subsets of outcomes due to limited number of studies.

## Discussion

The primary purpose of this meta-analysis was to determine whether tissue VEGF expression or serum VEGF level was associated with survival outcomes in NPC patients. We found that tissue high VEGF expression was associated with reduced OS and DFS in patients with NPC, while no survival associations were noted for other outcomes like RFS, MFS, or DFS. To investigate the inter-study heterogeneity for OS subset, we found that it may result from patient demographics, tissue VEGF expression or serum VEGF level determination method and different statistical analyses. Subgroup analyses were also performed in order to assess the impact of these factors on the HR for OS among NPC patients, and yielded consistent results. Furthermore, results also indicated that serum high VEGF level was significantly associated with reduced OS. Our data indicate that determination of tissue VEGF expression and serum VEGF level could be useful for predicting outcome in NPC patients, especially for patient OS.

As a member of the platelet-derived growth factor, VEGF could contribute to the proliferation of vascular endothelial cells, mitogenesis, and angiogenesis, suppression of dendritic cell maturation, increase in the permeability of the blood vessels, facilitating the leakage of vascular contents and thus providing extracellular matrix for vascular formation and endothelial cell migration, which play a crucial role for the development of cancer cells (58–60). Yang and colleges reported that tumor-secreted VEGF-B could significantly remodel tumor microvasculature, resulting in leaky vascular networks, providing good microenvironment for tumor cell invasion (61). It was also considered as an independent prognostic marker for cancer metastasis.

To the best of our knowledge, the present meta-analysis is the first study which systematically investigates the role of both tissue VEGF expression and serum VEGF level in NPC patients' prognosis. By involving more than 1,800 patients, the results of our meta-analysis may be more reliable than individual studies. However, there are some limitations to our meta-analysis. Firstly, different criteria were applied to determine tissue high/low expression of VEGF or serum high/low level of VEGF with heterogeneous cut off values among studies. In some cases, the percentage of VEGF-expressing cancer cells was applied for the definition of VEGF high expression group of patients; however, in other cases, an immunoreactive score was considered in terms of both the cell percentage and the intensity of the staining. Considering this kind of inter-study heterogeneity, we only included studies using immunohistochemistry on whole slides or tissue microarrays as VEGF expression-detecting method. Secondly, some of the included studies did not directly provide the survival estimates (HRs) used to be pooled in the meta-analyses. Consequently, we could only abstract and obtain the data through the digitalization of the Kaplan-Meyer survival curves, as previously reported (62, 63). This estimation process could have led to a certain bias. Therefore, the results should be interpreted with caution. Thirdly, although most of the included studies described patient adjuvant therapy including surgery followed by chemotherapy or radiotherapy, some of the cases (11 out of 13) did not report patients' postsurgical adjuvant therapy. This kind of inter-study heterogeneity in NPC treatment schedules might potentially have influenced the survival outcome. Fourthly, there was limited number of studies involved in the analyses for outcomes other than OS, so the conclusions for those outcomes should be interpreted with caution. Finally, heterogeneity in outcome definitions and reporting was also noted within the included studies, and we would naturally consider whether selective reporting of results was over stating the importance of tissue VEGF expression or serum VEGF levels in the progression of NPC.

In summary, this meta-analysis showed that high tissue VEGF expression significantly correlated with poor OS and DFS in NPC patients and high serum VEGF level was also significantly correlated with poor OS in NPC patients. Determination of tissue VEGF expression and serum VEGF level offers the potential to serve as biomarkers and add prognostic information in NPC. Larger sample-size prospective studies with a unanimous definition of the cut off level to detect VEGF expression or serum VEGF level are urgently required to advance our understanding of the relationship between VEGF and NPC outcomes.

## Author contributions

XL study concept and design. FW and LP acquisition of data. FW, LP, and YW analysis and interpretation of data. XL drafting of the manuscript. All authors critical revision of the manuscript for important intellectual content. XL study supervision.

### Conflict of interest statement

The authors declare that the research was conducted in the absence of any commercial or financial relationships that could be construed as a potential conflict of interest.
