# Supplementary material for: A Meta-Analysis of Vascular Endothelial Growth Factor for Nasopharyngeal Cancer Prognosis
Source: Front Oncol. 2018 Oct 31;8:486. doi: 10.3389/fonc.2018.00486 (PMC6220117; doi:10.3389/fonc.2018.00486)
Supplement: Supplementary file 1 [file Table_1.DOCX]

**Supplementary Material．**

**Pubmed Search Strategy**

| 1. "Nasopharyngeal Neoplasms"[Mesh] |
| --- |
| 1. (Nasopharynx OR nasophar* OR rhinophar* OR naso-phar* OR chonae) AND (Neoplasms OR carcinom* OR cancer* OR precancer* OR pre-cancer* OR neoplas* OR tumor* OR tumour* OR malignan* OR premalignan* OR pre-malignan*) [Title] |
| 1. NPC [Title] |
| 1. 1 OR 2 OR 3 |
| 1. "Vascular Endothelial Growth Factor A"[Mesh] |
| 1. "Vascular Endothelial Growth Factors"[Mesh] |
| 1. "Receptors, Vascular Endothelial Growth Factor"[Mesh] |
| 1. "Angiogenesis Inhibitors"[Mesh] |
| 1. (vascular endothelial growth factor or VEGF or VEGFR or FGFR or fibroblast growth factor or Bevacizumab or Aflibercept or Regorafenib) [Title] |
| 1. 5 OR 6 OR 7 OR 8 OR 9 |
|  |
| 1. 4 AND 10 |

**Embase Search strategy**

| 1. 'nasopharynx tumor'/exp |
| --- |
| 1. (Nasopharynx OR nasophar* OR rhinophar* OR naso-phar* OR chonae) AND (Neoplasms OR carcinom* OR cancer* OR precancer* OR pre-cancer* OR neoplas* OR tumor* OR tumour* OR malignan* OR premalignan* OR pre-malignan*): ti |
| 1. NPC:ti |
| 1. 1 OR 2 OR 3 |
| 1. 'vasculotropin a'/exp |
| 1. 'vasculotropin'/exp |
| 1. 'vasculotropin receptor'/exp |
| 1. 'angiogenesis inhibitor'/exp |
| 1. 5 OR 6 OR 7 OR 8 |
| 1. (‘vascular endothelial growth factor’ or VEGF or VEGFR or FGFR or ‘fibroblast growth factor’ or Bevacizumab or Aflibercept or Regorafenib):ti |
| 1. 9 OR 10 |
| 1. 4 AND 11 |

**Cochrane Library Search strategy**

| 1. MeSH descriptor: [Nasopharyngeal Neoplasms] explode all trees |
| --- |
| 1. (Nasopharynx or nasophar* or rhinophar* or naso-phar* or chonae) and (Neoplasms or carcinom* or cancer* or precancer* or pre-cancer* or neoplas* or tumor* or tumour* or malignan* or premalignan* or pre-malignan*):ti (Word variations have been searched) |
| 1. NPC:ti (Word variations have been searched) |
| 1. 1 OR 2 OR 3 |
| 1. MeSH descriptor: [Vascular Endothelial Growth Factor A] explode all trees |
| 1. MeSH descriptor: [Vascular Endothelial Growth Factors] explode all trees es |
| 1. MeSH descriptor: [Receptors, Vascular Endothelial Growth Factor] explode all trees |
| 1. MeSH descriptor: [Angiogenesis Inhibitors] explode all trees |
| 1. ‘vascular endothelial growth factor’ or VEGF or VEGFR or FGFR or ‘fibroblast growth factor’ or Bevacizumab or Aflibercept or Regorafenib:ti (Word variations have been searched) |
| 1. 5 OR 6 OR 7 OR 8 OR 9 |
| 1. 4 AND 10 |

**Supplementary Table 1. Definitions of 18 items of study reporting quality**

| Study design |
| --- |
| 1. Objectives or prespecified hypothesis: state the study objectives, prespecified hypothesis or study protocol |
| 2. Sample size: state a statistical sample size or power calculation |
| 3. Follow-up description: state the follow-up period or the median follow-up time |
| 4. Population source: state health care setting from which patients were recruited |
| 5. Population selection criteria: state inclusion or exclusion |
| 6. Population characteristics: state the population characteristics (e.g., age, gender, and disease stage) |
| 7. Number of patients included in each stage of the analysis and reason for dropout: description of number of patients at different stage, including the number of patients who participate in the study, who met the inclusion criteria, and who followed up and reason for dropout |
| Assay method |
| 1. Sample handling: state the method of storage |
| 2. Assay method: state the type of assay method used to measure VEGF |
| 3. Manufacturer: state the name of company which makes the assay for VEGF |
| 4. Cutoff point determination: state methods used for cutoff point determination |
| Confounders |
| 1. Conventional risk factors: state the conventional risk factors (e.g., age, gender, depth of tumor, lymph node metastasis) |
| 2. Other biomarkers (e.g., p53, PCNA, and microvessel density): state other biologic marker relating with the disease |
| Outcome |
| 1. Clinical endpoint: define the clinical endpoint |
| 2. Validation: state the outcome events checked by independent source (e.g., medical records, outpatient visits, by letter, and by telephone) |
| Analysis |
| 1. Univariate estimate: report the effect of VEGF on outcome |
| 2. Multivariate estimate: adjusted for risk factors or other biomarkers (list above) |
| 3. Missing value: state the number of patients with missing value for VEGF or confounders and how to deal with it |
